# Intimate partner violence: protocol of a quasi-experimental study to increase primary care professionals’ ability to identify exposed individuals

**DOI:** 10.1136/bmjopen-2024-095421

**Published:** 2026-05-24

**Authors:** Wibke Jonas, Karin Dahlström, Ragnhild Eikemo, Ylva Elvin Nowak, Uno Fors, Caroline Hurtig, Lene Lindberg, Margaretha Rhen, Terese Stenfors, Karolina Sörman, Mabel Zamora Hernandez, Mia Barimani

**Affiliations:** 1 Department of Women's and Children’s Health, Karolinska Institute, Stockholm, Sweden; 2 Academic Primary Care Centre, Region Stockholm, Stockholm, Sweden; 3 Institute of Clinical Sciences, Sahlgrenska Academy, University of Gothenburg, Gothenburg, Västra Götaland County, Sweden; 4 Department of Computer and Systems Sciences, University of Stockholm (Stockholm University), Stockholm, Sweden; 5 Department of Health, Medicine, and Caring Sciences, Linköping University, Linköping, Sweden; 6 Department of Global Public Health, Karolinska Institutet, Stockholm, Sweden; 7 Department of Learning, Informatics, Management and Ethics (LIME), Karolinska Institutet, Stockholm, Sweden; 8 Centre for Psychiatry Research, Department of Clinical Neuroscience, Karolinska Institutet, Stockholm, Sweden

**Keywords:** Health, Health Services, Health Impact Assessment

## Abstract

**Introduction:**

The overall aim of the present project is to increase healthcare professionals’ ability to ask about exposure and to identify individuals exposed to intimate partner violence (IPV). The project will evaluate the effects of three different interventions that can be assumed to increase healthcare professionals’ ability to ask and identify individuals who have been or are exposed to IPV.

**Methods:**

This project has a quasi-experimental design. After a 2-month baseline period, participating care units (primary health centres, maternal health clinics and youth guidance clinics) will be assigned to one of three interventions to potentially increase the ability to enquire and identify patient exposure to IPV: (1) healthcare professionals’ use of a standardised questionnaire about exposure to IPV in patient meetings, (2) training through the use of a virtual patient case tailored to health professionals and (3) a combination of (1) and (2) earlier. Preintervention (baseline) and postintervention measurements of the health professionals’ enquiry and identification of patients exposed to IPV will be used to explore the effect of the interventions. Focus group interviews with the participating health professionals will be used as a qualitative method, applying thematic analysis, to explore which intervention they perceive as most effective in increasing their ability to identify victims of IPV.

**Analysis:**

Data analysis will focus on a comparison of pre- and post-measurements regarding the number of patients asked about and identified patients in each intervention arm that have been or are exposed to IPV. Measurements will be carried out per care unit at the group level. Qualitative data from focus group interviews will be analysed using thematic analysis.

**Ethics and dissemination:**

All participants will sign a written consent form and the study has been approved by the Swedish Ethical Review Authority (Dnr 2023-03399-01). The study will be conducted according to good clinical practice and the Declaration of Helsinki. The results of this study will increase knowledge about how identification of violence in close relationships can be improved in the clinical setting through publications in peer-reviewed journals and presentations at national and international scientific conferences.

**Study status:**

Recruiting since May 2024. Expected trial termination December 2026.

**Trial registration number:**

NCT06322251.

**STRENGTHS AND LIMITATIONS OF THIS STUDY**

- The quasi-experimental design with pre- and post-measurements allows for assessments of changes over time and comparisons of different interventions, providing robust data on the effectiveness of each method.
- Our study fills a documented need to identify and evaluate useful and effective interventions that support clinical practice to identify exposure to intimate partner violence (IPV).
- The approach of using three distinct interventions (standardised questionnaire, training with virtual patients and a combination of both) is unique and allows for a comprehensive evaluation of how best to identify exposure to IPV.
- Our mixed-methods approach, combining quantitative data and qualitative insights from focus group interviews analysed using thematic analysis, provides a richer, more nuanced understanding of the interventions’ feasibility and their impact.
- The study will be conducted within specific healthcare settings in the Stockholm region, Sweden, which may limit the generalisability of the results to other regions or healthcare systems.

## Introduction

 Intimate partner violence (IPV) is widely recognised as a significant public health concern, with profound implications for affected individuals. Globally, nearly one-third of women have experienced IPV during their lifetime.^1^ The WHO defines IPV as ‘any behavior within an intimate relationship causing physical, sexual, or psychological harm, encompassing physical aggression, sexual coercion, psychological abuse, and controlling behaviors’.^2^ Most IPV incidents are directed towards women by male partners.^3^

### Psychological and physical sequelae of IPV

Extensive research from diverse cultural contexts has documented the multifaceted consequences of IPV exposure on the health of women. These consequences include chronic pain, functional gastrointestinal disorders, gynaecological problems, depression and anxiety symptoms and post-traumatic stress disorder.^4^,^9^ Notably, recent studies have shed light on the insidious nature of psychological IPV and its severe adverse health effects.^10^,^14^ Women who have experienced IPV often present to healthcare facilities and seek assistance for various and diffuse symptoms. The root cause of their health problems is often related to exposure to IPV, but this frequently goes undisclosed.^15 16^

### Screening of IPV

Many healthcare providers find it difficult and challenging to ask their patients or clients about potential IPV exposure, and in the case of a positive answer, they often do not know how to guide and help the client.^3 17 18^

Thus, despite the relatively high prevalence and adverse effects of IPV, IPV exposure tends to go unnoticed during healthcare visits. Consequently, timely interventions and support for affected women are hindered.^15 16^ Within healthcare settings, standardised questionnaires aimed at eliciting information about IPV exposure have been developed.^19^,^21^ However, there is ongoing debate regarding the effectiveness of universal screening for IPV. While the WHO^22^ clearly advises against routine enquiry in all healthcare encounters, international meta-analyses suggest that systematically asking patients about IPV, when assessing health conditions that are possibly linked to or complicated by IPV, can significantly increase the identification of victims.^17 18^ In the Swedish setting, it is not yet known whether the use of IPV questionnaires—an opportunity to ask patients/clients directly about potential IPV exposure—can be a helpful tool for healthcare professionals.

### Virtual patients

Despite the increasing demand for digital education solutions, studies exploring the use of virtual patients (VPs) in a primary care context in connection with IPV are lacking, presenting an opportunity for further investigation. VPs represent simulations of clinical scenarios with digital patient encounters. They can be used to train clinical reasoning and assessment, making them a valuable tool for healthcare professionals. VPs facilitate active learning and problem-solving while providing automatic feedback on patient management.^17^,^23^ Simulations with VPs are designed around problem-centred scenarios, engaging users in solving real-world-like problems. VPs may incorporate interactive features encompassing medical history taking, physical examination, laboratory tests and diagnostic and treatment plans.^24^ This approach not only activates long-term memory but also prompts the application of previous knowledge to generate new insights^25^ thereby potentially contributing to improved clinical reasoning and knowledge acquisition.^25 26^ VPs have been successfully used as a training arena based on other issues, including traumatised refugees.^26^ It is therefore useful to study whether VPs can also be used to train professionals to discover and manage IPV.

To summarise, the high incidence of psychosocial and physical health problems among people exposed to IPV may be associated with presentations at health services. Given the high prevalence of IPV and the associated medical consequences and costs, it is critical to address this public health problem. Our goal is to contribute tools to ask about and identify more individuals exposed to IPV.

## Objectives

The *overall aim* of this project is to increase primary care professionals’ ability to identify patients exposed to IPV. The *specific research questions* are: (1) to what extent do the following three interventions contribute to asking about and identifying potential victims of IPV: (a) the use of a standardised questionnaire to screen for exposure to IPV, (b) healthcare professionals’ training in IPV, using VPs tailored for clinical use and (c) a combination of interventions (a) and (b) earlier and (2) which of the three interventions do healthcare professionals experience as most effective to increase their ability to enquire about and identify and respond to victims of IPV?

## Methods and analysis

### Study design

**Figure 1:**
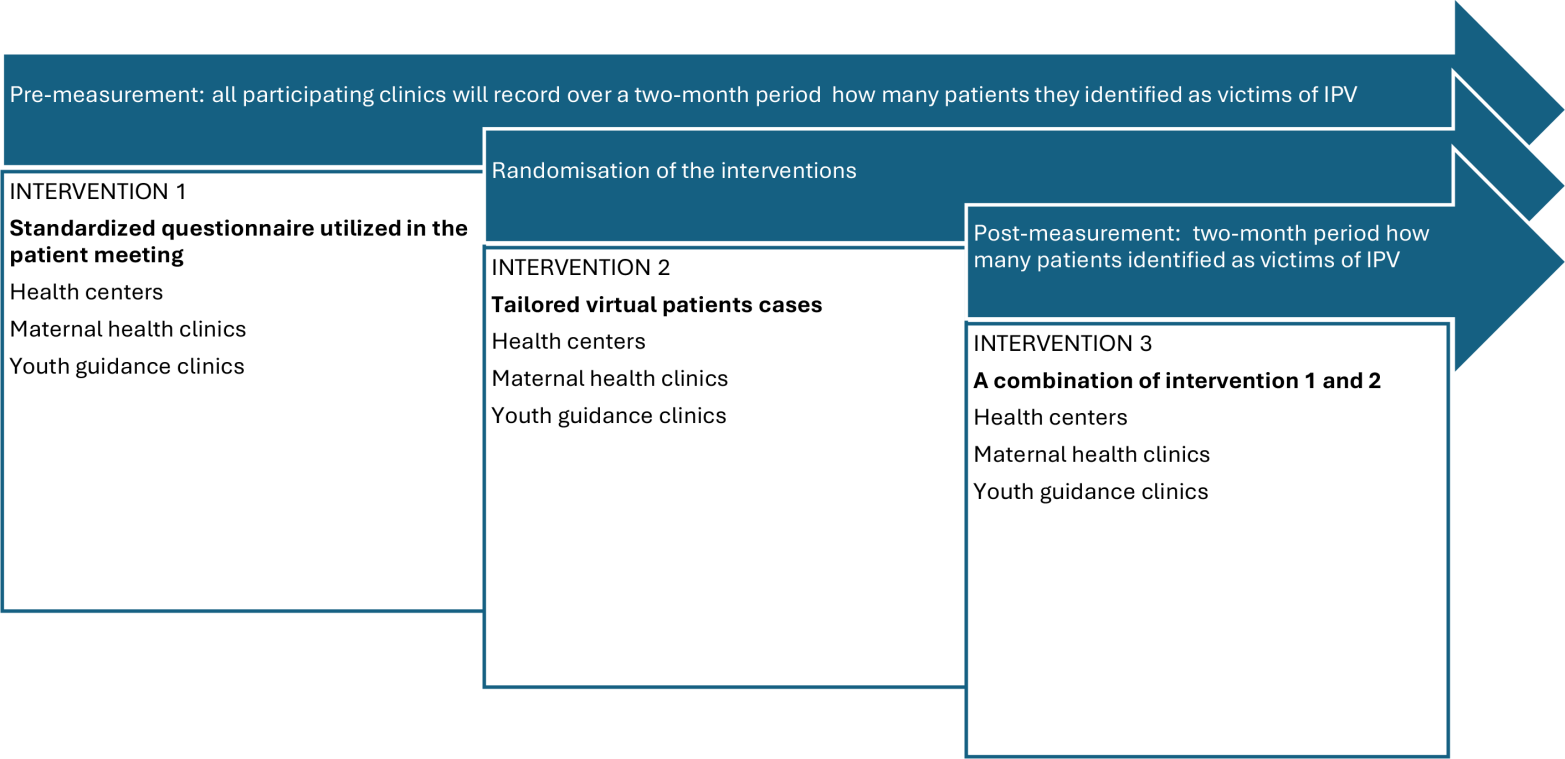
Overview of study design including the three interventions *standardised questionnaire*, *virtual patient* and *combination of the two*, as well as study settings. Preintervention and postintervention measurements of the number of identified patients exposed to IPV will be carried out at all study sites. IPV, intimate partner violence.

This study has a quasi-experimental design. After a baseline period, three interventions will be compared with evaluate their effects on healthcare professionals’ ability to ask and identify patients who are exposed to IPV. Three different types of care units will be included. These care units are primary health centres, maternal health clinics and youth guidance clinics, which will be assigned to deliver one of three interventions: (1) use of a standardised questionnaire for patients, (2) training using VPs tailored for healthcare professionals and (3) a combination of (1) and (2) above. Pre- and post-measurements of the health professionals’ identification of patients exposed to IPV will be used to explore the effect of the interventions. The focus group interviews with the healthcare professionals will be conducted as part of a qualitative methodological approach to complement the quantitative data. The trial is registered at ClinicalTrials.gov (NCT06322251). An overview of the design and interventions is shown in figure 1.

### Setting

The choice of healthcare units is based on the Swedish National Board of Health and Welfare’s mapping of important care units for identifying IPV.^27^ Accordingly, we will recruit health centres, maternal health clinics and youth guidance centres. *Health clinics* are primary healthcare facilities that provide a wide range of medical services to the public. These clinics serve as the first point of contact within the Swedish healthcare system and play a crucial role in maintaining public health. *Maternal health clinics*, also known as midwifery clinics, are specialised healthcare facilities that provide a wide range of services related to pregnancy, childbirth and postnatal care. These clinics are an essential part of Sweden’s healthcare system, ensuring the well-being of mothers and babies. *Youth guidance centres* are facilities that provide support and services specifically for young people, typically aged 13–25 years. These centres offer a wide range of services aimed at addressing young people’s health and well-being, including physical, mental and sexual health. There is no formal minimum age for patient inclusion in this study. In Sweden, youth guidance clinics routinely offer services to individuals from the age of 13, and it is standard practice to ask about exposure to violence in this age group. In our study, data from youth clinics will be collected through anonymised registry data, and according to the Swedish Ethical Review Authority, these individuals are therefore not considered study participants in the formal sense. While there are currently no standardised routines or structured questions for asking about IPV in this age group, our intervention aims to support and improve this practice.

### Population

#### Recruitment

The recruitment process differed for the three types of facilities, due to their number and size:

*Health centres* were informed about the study and invited to participate. As of January 2024, there were 235 healthcare units in Stockholm region. The inclusion process had two steps:

- Provider: units were included if they were run by the Stockholm County Health Department.
- Size: units with more than 8500 listed patients were included.

This resulted in 41 eligible units. In the second step, these units were ranked using a socioeconomic Care Need Index (CNI) to ensure diverse representation.This index, based on variables such as employment status, education level, age, single parenting and country of birth, was used. Invitations were sent to every second and third unit on the list (28 units in total), starting with the lowest socioeconomic index. Six health centres agreed to participate, and healthcare professionals consisted of general practitioners (GPs), district nurses and nurses.

*Maternal health clinics* were informed about the study and invited to participate. As of January 2024, there were 70 maternal health clinics in Stockholm region, and all were invited. Ten clinics agreed to participate, with midwives as the primary professionals.

*Youth guidance clinics* were informed about the study and invited to participate. As of January 2024, there were 29 youth guidance clinics in Region Stockholm, and all were invited. 11 clinics agreed to participate, with midwives as the primary professionals. Of note, there is no overlap in midwives’ employment at the maternal health and youth clinics.

### Procedures

#### Baseline assessments

Consenting healthcare professionals will receive information about the study. During a 2-month study period, baseline measurements will be performed.

The quasi-experimental design is based on pre- and post-measurements to assess the proportion of patients asked about possible exposure to IPV and identify persons exposed to IPV. Baseline measurement will be recorded (before randomisation to intervention) by all participating clinics over a 2-month period regarding how many patients they identify who have been exposed to IPV.

In terms of the *health clinics* and *youth guidance clinics*, data regarding the number of patients asked about IPV and the number of patients exposed to IPV will be retrieved from the medical record system Take Care, in which healthcare professionals document whether they have asked the patient about exposure to violence and what the patient has responded. This will be carried out by healthcare professionals as part of their regular documentation of the patient’s medical visit. Information about measures taken and the support given by the healthcare professional if the patient reports exposure will also be documented and retrieved from the same medical system.

Notably, in relation to *maternity clinics*, there are no diagnostic codes for exposure to IPV, and this information cannot thus be searched for in patients’ medical records. The research group has therefore developed a simple protocol in which healthcare professionals can record statistics on how many patients have been asked about possible exposure to IPV and how many patients have disclosed exposure to IPV during the study period.

#### Allocation of intervention

Health clinics, youth guidance clinics and maternity clinics will have separate allocations divided equally between the three interventions, together with a matching of socioeconomic status of the area where the clinic is located and the number of participants at the clinic (healthcare professionals). The allocation of healthcare units will be determined to avoid contamination between the three interventions, while the inclusion of socioeconomic status and the number of participants aims to balance the interventions. The healthcare units will be allocated separately by randomisation to intervention 1, 2 or 3.

The CNI will be used as a measure of socioeconomic status and is an instrument that is used to identify health risks at the area level using factors such as elderly people living alone, children below the age of 5, unemployment, low education, lone parents, relocation and migrants.^28^ The CNI will be coded as high or low, and the number of participants will be coded as two groups: many (>X?) or few (<X?). Computerised randomisation will be carried out using SPSS (version 29.0) during baseline. The clinics will be informed after baseline assessments.

## Interventions

Before the start of the interventions, the research team will provide the participating healthcare personnel with information.

*Intervention a* (figure 1)*:* a questionnaire will be used. The healthcare professionals will receive an overview of the Questionnaire about Intimate Partner Violence^29^ and how it should be administered to the patients. The IPV questionnaire contains 12 questions and measures exposure to physical, psychological and sexual violence over the past 12 months, which is the usual definition of ongoing exposure to IPV.

The opening question is: ‘Have you ever been exposed to psychological, physical or sexual violence from your partner or someone else close to you?’.

If the answer is ‘Yes’, ongoing exposure to partner violence is based on answers to the following questions: ‘During the past 12 months, have you been punched, kicked, pushed or injured by your partner or someone else close to you?’, ‘During the past 12 months, have you been subjected to psychological violence such as verbal abuse, threats or coercion by your partner or someone else important to you?’ and ‘During the past 12 months, have you been coerced into or subjected to sexual acts against your will by your partner or someone else important to you?’. Questions regarding the frequency of violence are included in the questionnaire with the answer options ‘Daily, Several times a week, Several times a month and Occasionally’. Finally, the patient will be asked whether they are currently afraid of a partner and whether they have children in the household. The closing question focuses on ever-in-life exposure, and is formulated: ‘If you have been exposed to intimate partner violence earlier in life, does this experience affect your health negatively today?’.

Healthcare professionals will be instructed to use these standardised questions over the 2-month study period (figure 1) during all patient visits to the healthcare facility, regardless of the reason for the visit. Some clinically relevant exceptions for asking patients will be added, for example, patient psychosis or other diagnoses that may affect the patient’s perception of reality, and situations where a patient is accompanied by someone who is presumed to be a perpetrator. Importantly, healthcare professionals will be asked only to use the standardised questions/ask about IPV exposure when the patient presents alone, to guarantee patient safety.

*Intervention b (figure 1):* in the current project, a tailored VP will be created for each of the three study settings (primary health centres, maternity clinics and youth guidance clinics). The VP cases are designed to mimic patient visits to a ‘virtual health centre’, during which the user can obtain background information about the fictitious patient, ask medical history questions, receive answers from the VP in the form of video clips and finally answer a few questions regarding the management of a case like the one presented. After this, at the end of the session with the VP, the user will receive feedback on their performance (1) from the VP themselves on how the visit felt in terms of, for example, trust, respect and treatment (including through video clips), (2) from a virtual expert on, for example, relevant questions asked, specific questions about IPV, information and talking about violence in the correct way and (3) on the answers asked relating to diagnosis, further management, etc. In this way, the healthcare professionals will practice how to formulate questions for patients and how responses could be interpreted, whether the patient needs care, and whether—and how—the patient should be referred to a psychiatrist or a psychologist, for example.

The VP cases are developed and run on a web-based platform called Virtual Case System developed by Stockholm University, Sweden. In the current project, tailored VP cases were developed for each of the three study settings (primary health centres, maternal health clinics and youth guidance clinics). ‘Tailored’ refers to the adaptation of the VP scenario to the clinical context of the respective setting to ensure relevance and realism. For example, the VP case for youth guidance clinics involves a young person seeking contraceptive advice, while the maternal health clinic VP case represents a pregnant woman attending her first antenatal visit. This approach aims to make the training applicable to the healthcare professionals’ everyday practice.

Finally, *intervention c* consists of a combination of *interventions a and b*, with both interventions described earlier being used to investigate identifying IPV over a 2-month period after the VP training has been carried out and in parallel with the use of the Questionnaire about Intimate Partner Violence.

### Intervention assessment

During the intervention period, the identification of patients asked about and exposed to IPV will be assessed in the same way as described earlier.

### Outcome measures

*Primary outcome:* the primary outcome is the number of patients asked about IPV and patients identified exposed to IPV. This will be investigated before (baseline) and after the intervention period. Data will be measured at group level within all participating healthcare units (healthcare centres, maternal health centres and youth guidance clinics). Measurements will take place per care unit (at group level), and the analyses will mainly be comparisons of pre- and post-measurements.

The estimated number of patient visits reflects the total volume at participating units during the study period. The study does not analyse individual patient data beyond whether IPV was asked about and disclosed. Each unit will include its usual patient population, meaning youth guidance clinics will include adolescents, including those under 18 years of age. Screening will be conducted according to local protocols and ethical approval requirements.

It is important to note that the lifetime prevalence of IPV, often reported in global estimates, differs substantially from past-year prevalence. In this study, the screening tool focuses on exposure within the past 12 months, as this timeframe is most relevant for identifying individuals currently at risk and in need of clinical intervention. Consequently, the number of cases detected through screening will be lower than lifetime prevalence figures.

As specified in the research questions, the aim of this study is to evaluate interventions that improve healthcare professionals’ ability to ask about IPV and identify exposed individuals. The study does not analyse health outcomes or long-term health effects of IPV. The standardised questionnaire described is used solely as part of the intervention to facilitate enquiry and identification, not for collecting or analysing clinical health data. The primary outcome is limited to the number of patients asked about IPV and the number of patients who disclose exposure during the study period.

*Qualitative outcomes:* in addition to the quasi-experimental design, the study includes a qualitative component aimed at exploring healthcare professionals’ experiences of the interventions. This part of the study is based on a qualitative interpretative design using thematic analysis.^30^ Focus group interviews^31^ will be conducted with participants from each care unit type (primary health centres, maternal health clinics and youth guidance clinics), representing all three intervention arms. The interviews will be transcribed verbatim and analysed inductively to identify patterns and themes related to perceived effectiveness, feasibility and clinical relevance of the interventions. This qualitative approach is intended to complement the quantitative findings and provide a deeper understanding of the interventions’ impact in clinical practice.

To better understand and meaningfully explain the results, and to gain insight into healthcare professionals’ attitudes and behaviours, all study participants will be offered the opportunity to participate in focus group interviews after the analysis work from the interventions has been completed. Each care unit (health centres, maternal health clinics and youth guidance centres) will participate in an individual focus group, meaning that there will be healthcare professionals representing the different interventions in each focus group. In these groups, the focus will be on participants’ own experiences of how effective each intervention has been in identifying patients exposed to IPV. This will be an important complement to the above quantitative analyses and will inform clinical practice. We are planning to conduct six focus groups with four to six healthcare professionals in each group. Demographic data from study participants such as age, education and profession will also be collected. The qualitative component of the study is based on an interpretative design using thematic analysis^30^ and focus group interviews are employed as the method for data collection.^31^

*Sample size calculation:* at the health centres, the healthcare professionals are particularly represented through GPs and district nurses. At the maternal health centres and youth guidance clinics, midwives are the main profession represented. We estimate that approximately 60 healthcare professionals will take part in each intervention arm (figure 1). GPs and midwives meet approximately seven to ten patients per day, a number that may vary depending on healthcare professionals working full- or part-time. However, we estimate that there is a sufficiently large base for a quasi-experimental design (ie, a preintervention and postintervention measurement with comparisons between the interventions), where the main outcome measures are the number of asked and identified persons exposed to IPV. Thus, approximately 6000 patients will be approached in each intervention arm during the study period. With a reported national prevalence of IPV of 7%, 420 cases of IPV could potentially be identified in every study arm. It should be noted that the 7% prevalence of IPV is based on a national sample and may vary in the study setting. These numbers are estimated to be large enough to detect a medium effect size with a power of 0.80 and an α-level of 0.05.

## Data management

Data will be stored on a secure server at Region Stockholm that can only be accessed by research team members who work actively with data analysis. Data will be pseudonymised. Interview recordings will be destroyed as soon as the data have been transcribed.

### Data analysis and statistics

#### Quantitative analysis

Data will be analysed at group level within each study setting (health centres, maternal health clinics and youth guidance centres). The analysis will primarily involve pre- (baseline) and post-intervention assessments, comparing the number of patients asked about IPV and identified of IPV exposure during baseline and after the intervention period using logistic regression analyses (with patients asked about IPV and patients identified with IPV exposure as outcomes and time (pre and post) and intervention arms and a time×intervention interaction term as predictors. Covariates will be used when relevant.

### Qualitative data

In addition to the quasi-experimental design, the study includes a qualitative component aimed at exploring healthcare professionals’ experiences of the interventions. This part of the study is based on a qualitative interpretative design using thematic analysis.^30^ Focus group interviews^31^ will be conducted with participants from each care unit type (primary health centres, maternal health clinics and youth guidance clinics), representing all three intervention arms. The interviews will be transcribed verbatim and analysed inductively to identify patterns and themes related to perceived effectiveness, feasibility and clinical relevance of the interventions. This qualitative approach is intended to complement the quantitative findings and provide a deeper understanding of the interventions’ impact in clinical practice.

### Ethics and dissemination

The project has received ethical approval (Dnr 2023-03399-01) and complies with the Declaration of Helsinki. Ethical considerations include potential triggers for health professionals who have experienced violence themselves. In the event of such an incident, a psychologist or psychotherapist research team member with IPV competence will provide support and guidance. Further, sensitive situations may arise if attitudes about people exposed to IPV are discussed in focus groups. Considering this, study participation is voluntary and can be discontinued at any time. One ethical question concerns what the additional value of the study is for individuals exposed to IPV. We believe that the study will contribute new knowledge about how health professionals can ask, identify and respond to IPV exposure to these patients and thus, in the longer term, we believe this project can contribute to an increased ability to offer support to individuals exposed to IPV. The project is likely to result in an increase in the number of individuals asked about IPV when visiting primary care. Previous research indicates that both victims and non-victims appreciate being asked about violence by healthcare professionals. Screening for IPV in this study is conducted within healthcare units that have established local protocols for responding to disclosures of violence. Prior to inclusion, all participating units confirmed access to resources and referral pathways for appropriate support. Ethical approval was granted on the basis that these safeguards are in place, ensuring that screening does not occur in isolation but as part of a structured response system.

The results will be disseminated to the scientific community through articles and at conferences. Clinical healthcare professionals will be educated about the study results, and student curricula will be updated. The results will be disseminated to society via social media, magazines and newspapers.
