# Supplementary material for: Intimate partner violence: protocol of a quasi-experimental study to increase primary care professionals’ ability to identify exposed individuals
Source: BMJ Open. 2026 May 24;16(5):e095421. doi: 10.1136/bmjopen-2024-095421 (PMC13202110; doi:10.1136/bmjopen-2024-095421)
Supplement: Supplementary file 1 [file bmjopen-2024-095421.reviewer_comments.pdf]

PEER REVIEW HISTORY

BMJ Open publishes all reviews undertaken for accepted manuscripts. Reviewers are asked to complete a checklist review form and are provided with free text boxes to elaborate on their assessment. These free text comments are reproduced below.

ARTICLE DETAILS

Title (Provisional)

Intimate partner violence: protocol of a quasi-experimental study to increase primary care professionals' ability to identify exposed individuals

Authors

Jonas, Wibke; Dahlström, Karin; Eikemo, R.; Elvin Nowak, Ylva; Fors, Uno; Hurtig, Caroline; Lindberg, Lene; Rhen, Margaretha; Stenfors , Terese; Sörman, Karolina; Hernandez, Mabel; Barimani, Mia M.

VERSION 1 - REVIEW

**Reviewer**	**1**
**Name**	**Macdonald, Marilyn**
**Affiliation**	**Dalhousie University**
**Date**	**30-Jan-2025**
**COI**	**None**

Thank-you for the opportunity to review this paper on an important and timely topic.

Title – states a protocol for a quasi-experimental study.

Abstract – methods state "Focus group interviews with the participating health professionals will be a qualitative complement, in terms of which intervention they experience as the most effective in increasing their ability to identify victims of IPV".

Focus groups is a methodology as well as method.

Analysis – states "focus group interviews will be analyzed using thematic analysis". (Braun & Clark. 2012).

Strengths and limitations bullet 4 states "Our mixed-methods approach, with a combination of quantitative data and qualitative insights from focus group interviews, provides a richer, more nuanced understanding of the interventions' feasibility and their impact".

Page 3, line 52, typo, remove 'be'

Methods and analysis header – addresses the quasi-experimental design with no identification of the design of the qualitative dimension of the project.

The qualitative design needs to be identified and described. For example, if the design is Thematic analysis, please refer to Braun & Clark (2022), Thematic Analysis a practical guide. TA is both methodology (design) and method. Focus Group is also Methodology and method Kruger & Casey (2015), Focus Groups 5th edition A practical guide for applied research.

Will participants be interviewing women of all ages and genders?

**Reviewer**	**2**
**Name**	**Gulliver, Pauline**
**Affiliation**	**University of Auckland, School of Population Health**
**Date**	**21-Aug-2025**
**COI**	**None**

I am struggling with the theoretical basis for this investigation. Firstly, the prevalence estimate quoted is lifetime prevalence. This will be substantially different from past-year prevalence and therefore the number of women who could be expected to be identified through a screening tool for experiencing violence currently (which I am assuming is what is going to be screened for). Secondly, the health impacts quoted are generally developed over a lifetime, so there can be a long lag period between when the violence was experienced and the development of the health issue. Has this been considered? Thirdly, screening alone won't support the provision of an appropriate response and if the health professional does not have access to resources or support agencies to provide that response, then screening on its own is likely to be counterproductive.

Page 5, is there an overlap between the midwives working in the maternity clinics and those working in the youth guidance clinics? How is this accounted for?

- I am concerned about the self-report component for midwives. What quality control mechanisms are in place?

Page 6: I expect there is a minimum age for patient inclusion in this investigation. Please include this.

Page 7, how do you know if the health professional takes part in the VP training?

- I also am not clear on what a 'tailored VP' is - does this mean that you are only using patients with particular characteristics?

- the statistical approach has not been adequately described.

Page 8, pregnancy is not protective against IPV.

- 6000 patients likely also includes children? Are they going to be screened for IPV?

- I don't imagine midwifery is included in this 6000 estimate. Appointments with midwives can take a lot longer than standard consultations.

VERSION 1 - AUTHOR RESPONSE

**Reviewer 1**	
Title – states a protocol for a quasi-experimental study.	
Abstract – methods state "Focus group interviews with the participating health professionals will be a qualitative complement, in terms of which intervention they experience as the most effective in increasing their ability to identify victims of IPV". Focus groups is a methodology as well as method.	We agree that focus groups represent both a method and a methodology. In our study, focus group interviews are used as a qualitative method to complement the quantitative findings, with the aim of capturing participating healthcare professionals' experiences of the effectiveness of the interventions. We have now clarified this in the **Methods section and the Abstract** and added information about the qualitative analysis approach.<br><br>**The text now reads (abstract, page 2):** "Focus group interviews with the participating health professionals will be used as a qualitative method, applying thematic analysis, to explore which intervention they perceive as most effective in increasing their ability to identify victims of IPV."<br><br>**Methods (page 6):** "The focus group interviews with the health care professionals will be conducted as part of a qualitative methodological approach complementing the quantitative data."
Analysis – states "focus group interviews will be analyzed using thematic analysis". (Braun & Clark. 2012). Strengths and limitations bullet 4 states "Our mixed-methods approach, with a combination of quantitative data and qualitative insights from focus group interviews, provides a richer, more nuanced understanding of the interventions' feasibility and their impact".	We appreciate the opportunity to clarify. We have now updated the manuscript to reflect this methodological choice more clearly.<br>In our study, focus group interviews will be analyzed using thematic analysis, as described by Braun and Clarke (2022). This approach allows for a flexible and in-depth exploration of healthcare professionals' experiences and perceptions of the interventions. Thematic analysis supports the

	development of rich, nuanced insights, which is the basis for the qualitative contribution (also mentioned in the Strengths and Limitations section). For a new text, please see **Analysis section, Qualitative data, page 12.**<br><br>**Strengths and limitations, bullet point 4 now states:** Our mixed-methods approach, combining quantitative data and qualitative insights from focus group interviews analyzed using thematic analysis, provides a richer, more nuanced understanding of the interventions' feasibility and their impact.
Page 3, line 52, typo, remove 'be'<br><br>Methods and analysis header – addresses the quasi-experimental design with no identification of the design of the qualitative dimension of the project.	Thank you, we have now removed the "be".<br><br>Thank you, we have now added and changed the qualitative aspects of the project. **The text now reads, page 12:** " In addition to the quasi-experimental design, the study includes a qualitative component aimed at exploring healthcare professionals' experiences of the interventions. This part of the study is based on a qualitative interpretative design using thematic analysis (Braun & Clarke, 2022). Focus group interviews (Krueger & Casey, 2015) will be conducted with participants from each care unit type (primary health centers, maternal health clinics, and youth guidance clinics), representing all three intervention arms. The interviews will be transcribed verbatim and analyzed inductively to identify patterns and themes related to perceived effectiveness, feasibility, and clinical relevance of the interventions. This qualitative approach is intended to complement the quantitative findings and provide a deeper understanding of the interventions' impact in clinical practice.

	**We have changed the Braun and Clarke 2012 citation to**: Braun, V., & Clarke, V. (2022). *Thematic Analysis: A Practical Guide*. London: SAGE Publications.<br><br>**We have also added:**<br>Krueger, R. A., & Casey, M. A. (2015). *Focus Groups: A Practical Guide for Applied Research* (5th ed.). Thousand Oaks, CA: SAGE Publications.
The qualitative design needs to be identified and described. For example, if the design is Thematic analysis, please refer to Braun & Clark (2022), Thematic Analysis a practical guide. TA is both methodology (design) and method. Focus Group is also Methodology and method Kruger & Casey (2015), Focus Groups 5th edition A practical guide for applied research.	Thank you for your insightful comment. We agree that both thematic analysis and focus groups represent methodological as well as methodical choices. In response, we have clarified the qualitative design in the "Methods and analysis" section, **page 13:**<br><br>"The qualitative component of the study is based on an interpretative design using thematic analysis (Braun & Clarke, 2022) and focus group interviews are employed as the method for data collection (Krueger & Casey, 2015)."<br><br>These additions ensure that both the methodological and analytical foundations of the qualitative dimension are clearly described (see answer to the comment above).

**Reviewer 2**	
I am struggling with the theoretical basis for this investigation.<br><br>Firstly, the prevalence estimate quoted is lifetime prevalence. This will be substantially different from past-year prevalence and therefore the number of women who could be expected to be identified through a screening tool for experiencing violence currently (which I am assuming is what is going to be screened for).	 <br><br>Thank you for highlighting this important point. We agree that lifetime prevalence differs substantially from past-year prevalence. In our study, the screening tool is designed to identify individuals with recent or ongoing exposure to IPV, defined as within the past 12 months. Therefore, the number of cases detected through screening will likely be lower than lifetime prevalence estimates. We have clarified this distinction in the

	manuscript by specifying that the questionnaire focuses on past-year exposure, which is the relevant timeframe for current risk and clinical intervention.<br><br>**The text now reads, page 12 (section outcome measures): "**It is important to note that lifetime prevalence of IPV, often reported in global estimates, differs substantially from past-year prevalence. In this study, the screening tool focuses on exposure within the past 12 months, as this timeframe is most relevant for identifying individuals currently at risk and in need of clinical intervention. Consequently, the number of cases detected through screening will be lower than lifetime prevalence figures."
Secondly, the health impacts quoted are generally developed over a lifetime, so there can be a long lag period between when the violence was experienced and the development of the health issue. Has this been considered?	As outlined in the research questions, the study exclusively aims to evaluate interventions that improve healthcare professionals' ability to ask about IPV and identify exposed individuals. No health outcomes or long-term health effects will be analyzed. The questionnaire is used solely as part of the intervention to facilitate inquiry and identification.<br><br>**We have clarified this in the text, see page 12, outcome measures:**<br>"As specified in the research questions, the aim of this study is to evaluate interventions that improve healthcare professionals' ability to ask about IPV and identify exposed individuals. The study does not analyze health outcomes or long-term health effects of IPV. The standardized questionnaire described is used solely as part of the intervention to facilitate inquiry and identification, not for collecting or analyzing clinical health data. The primary outcome is limited to

	the number of patients asked about IPV and the number of patients who disclose exposure during the study period.”
Thirdly, screening alone won't support the provision of an appropriate response and if the health professional does not have access to resources or support agencies to provide that response, then screening on its own is likely to be counterproductive.	Thank you for raising this important ethical consideration. We fully agree that screening without an appropriate response could be counterproductive. This issue has been addressed in the study design and ethical approval process. The study was approved by the Swedish Ethical Review Authority (Dnr 2023-03399-01) based on evidence that all participating healthcare units have established local protocols for managing IPV cases. Before inclusion, we verified that each unit has access to resources and referral pathways to support individuals who disclose violence. Thus, screening is embedded within a system that ensures appropriate follow-up and support.<br><br>**We have added the following text to the Ethics section, p 14/15**: “Screening for IPV in this study is conducted within healthcare units that have established local protocols for responding to disclosures of violence. Prior to inclusion, all participating units confirmed access to resources and referral pathways for appropriate support. Ethical approval (Dnr 2023-03399-01) was granted on the basis that these safeguards are in place, ensuring that screening does not occur in isolation but as part of a structured response system.”

Page 5, is there an overlap between the midwives working in the maternity clinics and those working in the youth guidance clinics? How is this accounted for?<br><br><br><br><br><br><br>- I am concerned about the self-report component for midwives. What quality control mechanisms are in place?	Thank you for your question. There is no overlap between midwives working in maternity clinics and those in youth guidance clinics. These facilities are separate and operate independently. We have added the following clarification on **page 8**: "Of note, there is no overlap in midwives' employment between maternal health and youth clinics."<br><br>Thank you for your comment. We acknowledge that no formal monitoring or verification of adherence to the intervention protocol is conducted. However, all midwives participating received detailed written and verbal instructions regarding the implementation of the intervention. We rely on the professionalism and clinical judgment of the healthcare staff and trust that they will follow the protocol as instructed. This approach reflects the real-world conditions of clinical practice and is consistent with the pragmatic nature of the study design.
Page 6: I expect there is a minimum age for patient inclusion in this investigation. Please include this.	Thank you for your comment. **We have added the following text (page 7)**: "There is no formal minimum age for patient inclusion in this study. In Sweden, youth guidance clinics routinely offer services to individuals from the age of 13, and it is standard practice to ask about exposure to violence in this age group. In our study, data from youth clinics will be collected through anonymized registry data, and according to the Swedish Ethical Review Authority, these individuals are therefore

	not considered study participants in the formal sense. While there are currently no standardized routines or structured questions for asking about IPV in this age group, our intervention aims to support and improve this practice.
Page 7, how do you know if the health professional takes part in the VP training?<br><br>I also am not clear on what a 'tailored VP' is - does this mean that you are only using patients with particular characteristics?<br><br>-	Thank you for your comment. We appreciate the opportunity to clarify.<br><br>The virtual patient (VP) training is delivered via a web-based platform that includes a built-in system for tracking user activity. Therefore, it is technically possible to monitor whether individual healthcare professionals complete the training. However, in line with the pragmatic nature of the study and its real-world clinical setting, we will rely on their professionalism and motivation to engage with the training as intended.<br><br>Thank you for your question. In this study, the term “tailored virtual patient (VP)” refers to the fact that each VP case has been specifically designed to reflect the clinical context of the respective study arm. For example, in the youth guidance clinic setting, the VP scenario involves a young person seeking contraceptive advice, while in the maternal health clinic setting, the VP represents a pregnant woman attending her first antenatal visit. These tailored cases aim to ensure relevance and realism for the participating healthcare professionals, thereby enhancing the applicability of the training to their everyday clinical practice. This does not mean selecting patients with a particular characteristic but rather creating realistic scenarios for each care setting.”<br><br>**We have added the following text on VP’s, page 11:**<br>“In the current project, tailored VP cases were developed for each of the three

the statistical approach has not been adequately described.	study settings (primary health centers, maternal health clinics, and youth guidance clinics). "Tailored" refers to the adaptation of the VP scenario to the clinical context of the respective setting to ensure relevance and realism. For example, the VP case for youth guidance clinics involves a young person seeking contraceptive advice, while the maternal health clinic VP case represents a pregnant woman attending her first antenatal visit. This approach aims to make the training applicable to the healthcare professionals' everyday practice."<br><br>**We have elaborated on the statistical approach on page 13:**<br>**Data analysis and statistics**<br>*Quantitative analysis*<br>Data will be analyzed at group level within each study setting (health centers, maternal health clinics, and youth guidance centers). The analysis will primarily involve pre- (baseline) and post-intervention assessments, comparing the number of patients asked about IPV and identified of IPV exposure during baseline and after the intervention period using Logistic regression analyses (with patients asked about IPV and patients identified with IPV exposure as outcomes and time (pre-and post) and intervention arms and a time x intervention interaction term as predictors. Covariates will be used when relevant.
Page 8, pregnancy is not protective against IPV.	Thank you for pointing this out. We agree that pregnancy is not protective against IPV. To avoid misinterpretation, we will remove the sentence suggesting lower IPV prevalence during pregnancy. The manuscript already states that prevalence may vary, which is sufficient for the context.

6000 patients likely also include children? Are they going to be screened for IPV?	Thank you for raising this point. We have clarified that the estimated number of patients (approximately 6,000 per intervention arm) refers to all patient visits at participating units during the study period. The study does not focus on the patients themselves but on the outcome measure at group level: the number of patients asked about IPV and those who disclose exposure. Each healthcare unit will include the patient population they normally serve, which means youth guidance clinics will include adolescents, including those under 18 years of age. Screening will follow existing local protocols and ethical approval requirements.<br><br>**We have also added the following text on p 12:**<br>"The estimated number of patient visits reflects the total volume at participating units during the study period. The study does not analyze individual patient data beyond whether IPV was asked about and disclosed. Each unit will include its usual patient population, meaning youth guidance clinics will include adolescents, including those under 18 years of age. Screening will be conducted according to local protocols and ethical approval requirements."
I don't imagine midwifery is included in this 6000 estimate. Appointments with midwives can take a lot longer than standard consultations.	**Thank you for this comment**. To clarify, in Sweden, visits at the midwifery clinic are scheduled to last 30 minutes. This is apart from the first visit during pregnancy, which is scheduled to last for 90 minutes.<br>**We have added the following text, page 8: "**Of note, there is no overlap in

	terms of midwives employment at the maternal health and youth clinics.”

VERSION 2 - REVIEW

**Reviewer**	**2**
**Name**	**Gulliver, Pauline**
**Affiliation**	**University of Auckland, School of Population Health**
**Date**	**13-Apr-2026**
**COI**	

The authors have addressed the concerns that I raised previously.
